# TARGETING IL-23: INSIGHTS INTO THE PATHOGENESIS AND THE TREATMENT OF PSORIASIS

**DOI:** 10.4103/0019-5154.62760

**Published:** 2010

**Authors:** Hermenio Cavalcante Lima, Alexandra Boer Kimball

**Affiliations:** *From the Department of Medical Pathology, Federal University of Parana - Curitiba, PR - Brazil*; 1 *From the Department of Dermatology, Clinical Unit for Research Trials in Skin - Massachusetts General Hospital, Harvard Medical School – Boston, MA – USA.*

**Keywords:** *Anti-IL-12/23*, *biological treatment*, *IL-12*, *IL-17*, *IL-23*, *psoriasis*

## Abstract

Therapeutic experience strongly supports the use of TNF antagonists as important modalities in the treatment of psoriatic arthritis and plaque psoriasis. Studies with anti-IL-12/23 therapeutic agents, which act in different steps of the psoriatic inflammatory cascade, have also shown demonstrable efficacy. Here, we discuss this approach and its potential within the armamentarium for the treatment of psoriasis. Evidences that the selective blocking of IL-23 may be effective and safe therapy are also addressed.

## Introduction

Blocking specific cytokines is a modern treatment option for psoriasis and other autoimmune diseases. There is a simple rational for these therapies. The uncontrolled production of some cytokines causes immune system dysregulation and subsequent pathology manifesting in multiple medical conditions. Interleukin (IL)-23, like many other cytokines, has an important role in health and disease.[1] Identified initially from human DNA sequence information, IL-23 is a stimulus for cell-mediated immune responses.[2] This review discusses the pivotal role of this cytokine in psoriasis pathophysiology, how our understanding of its mechanism evolved and how blocking its effect might substantially improve the condition of people with this disabling skin disease.

## Pre-Biologic Immunological History of Psoriasis

Biologic therapy for psoriasis initially derived from the evolution of concepts about its etiopathogenesis. As in a chess game, these new forms of treatment have evolved from an integration of the knowledge about how both the immune system cells (pieces) and its cytokines (movements) generate the immunophysiopathology of that manifests as psoriasis.

In ancient records, the initial causes of psoriasis were attributed to multiple sources ranging from the divine to racial associations at the end of the 19th century.[3] An unknown infectious organism was identified as a source of psoriasis in 1927.[4] Later, its etiology was described as primarily and essentially an epidermal problem, independent of immunologic phenomena[5] The main objective of the cytotoxic drugs, such as methotrexate, developed in the 20th century was to reduce keratinocyte proliferation. Immunological studies on psoriatic patients indentified changes in humoral immune reactions as part of the overall problem but not the cause.[6,7] Eventually, other studies identified that the action of cytotoxic drugs extended to the immune system of patients with psoriasis.[8,9] However, the dominant thought was that psoriasis was a disease of faulty epidermopoiesis due to impaired autocontrol mechanisms.[10] Hunter *et al.* wrote “More work on cell turnover and its regulation will give the clue to psoriasis.”[11]

Further investigations in the 1970s revealed the role of immunologic factors in psoriasis. Under histopathologic examination, psoriatic lesions showed a striking resemblance to cellular inflammatory reactions observed in areas affected by contact allergic dermatitis.[12] A selective immunosuppressant effect was the initial hypothesis used to describe the a pathological cellular immune response.[13] Soon thereafter, the finding of a soluble factor that played a important role in keratinocyte proliferation and therefore induction/maintenance of psoriatic lesions triggered a more comprehensive cytokine-based theory.[14] At this point, the rapid turnover in the epidermis, genetically determined, but triggered by immunological factors emerged as a dominant integrated theory explaining the etiopathogenesis of psoriasis.[15]

The fundamental confirmation that any defect of the skin is not sufficient by itself to maintain a psoriatic lesion occurred in the subsequent decade. Some studies confirmed that T-cells and soluble factors could stimulate keratinocyte proliferation. Immunophenotyping of psoriatic lesions showed a mixed T-cell population (CD4 and CD8) and Langerhans cells distinct from normal skin.[16] This cellular infiltrate changed with topic or systemic treatment.[17,18] In another study, biologic therapy failure ruled out the major participation of the humoral immune system in psoriasis immunopathology.[19] Thus, the cellular arm of the immune system was implicated in psoriasis for the first time during the 1980s.[20]

Two main branches of research on immunogenesis of psoriasis, the cellular and the cytokine, developed during the 1990s. Researchers observed that an influx of activated, mainly CD4+, HLA-DR+, IL-2 receptor-CD25+, T-cells was one of the earliest events of psoriasis.[21] Based on Mosmman and Coffman's publication,[22] these T-cells were classified as type 1 cytokine producers.[23] They produce interferon (IFN)-γ, IL-2, and tumor necrosis factor (TNF)-α cytokines and therefore implied that a cellular type 1 of reaction was responsible for psoriasis.

## The Initial Biologic Treatments for Psoriasis and its Implications on the Understanding of the Psoriasis Immunological Mechanism

The primary etiopathogenesis of an autoimmune disease is dysregulation of immune system activation since the development of autoreactive lymphocyte occurs in the same basic manner as lymphocyte activation. Cytokines are key players in the establishment of any immune system reaction and therefore blocking cytokines that maintain autoimmune activity has become one the most successful strategies for autoimmunity therapy.

Psoriasis was defined as Th1 type of disease early in the understanding of the T-helper subsets. As a result, two logical biologic therapeutic approaches were tested: one was the administration of counter regulatory type 2 cytokines and the second was the blocking of type 1 cytokines. The use of monoclonal antibodies or fusion proteins to neutralize the Th1 cytokines started to be used in large scale because of their efficacy and practicability.

These studies have proved to be a useful biological model and test ground for evaluation of the skin immune system and psoriasis. Although these drugs were not initially developed in the treatment of psoriasis, but rather in rheumatoid arthritis and Crohn's disease, the observation that condition of Crohn's disease patients with psoriasis improved while on anti-TNF therapy profoundly influenced the studies that were to come. Moreover, this empiric observation enabled the research demonstrating that TNF was a key cytokine position in psoriasis etiopathogenesis by modulating the immunologic microenvironment in psoriatic skin.[24]

Although clinical response to anti-TNF suggested a role for Th1 cells in psoriases, evidence coming from other studies demonstrated that Th1/Th2 paradigm and TNF key role were not sufficient to explain the full etiopathogenesis of psoriasis. At this point, some academic resistance to an immunological pathogenesis for psoriasis could still be found in discussions of the immunology.[25] However, the main interpretation was that an important piece of the immunological cytokine puzzle was missing.

## The IL-12/23 Psoriasis Immunopathogenesis

The initial quest for the missing cytokines was the search for pathway inducers. Researchers first noted that IL-12 is crucial for Th1-cell differentiation.[26] IL-12 signaling via its receptor activates Stat4 (signal transducer and activator of transcription 4), which upregulates IFN-γ. IFN-γ activates Stat1, which enhance T-bet (T-box expressed in T-cells), the leading TH1 transcription factor, further enhancing IFN-γ production and downregulating IL-4 and IL-5 expression.[27] IFN-γ mediates many of the pro-inflammatory activities of IL-12. Phagocytes and dendritic cells (DCs) are the main producers of IL-12 in response to microbial stimulation,[28] and relationship links innate resistance and adaptive immunity. Its main function is resistance to infections with bacteria and intracellular parasites. However, it plays an important role in the Th1 response that sustains organ-specific autoimmunity.[29] The use of anti-IL-12 mAb(monoclonal antibody) in the experimental model of psoriasis also suggested the therapeutic value of blocking IL-12 in humans,[30] although side effects of the drug limited further development in this area.

For many years, the IL-12-dependent Th1 cells were thought to be essential for the induction of autoimmunity. However, during the Th1/Th2 paradigm studies, an IFN-γ-independent mechanism responsible for the pathogenesis of many inflammatory diseases and psoriasis was found.[31] The use of anti-IL-12/23p40 and anti-IFN mAb ultimately established at least part of the solution to the riddle. Only neutralization of p40, not IFN-γ, ameliorated chronic inflammatory reactions. IL-12 and IL-23, as discovered previously from human DNA sequence information, share the subunit p40.[32] This finding therefore suggested that the latter cytokine accounted for the IFN-γ-independent mechanism of inflammation.

Identified from human DNA sequence information, IL23, like IL-12, is also a heterodimeric cytokine composed of the same subunit p40 paired with the unique p19.[2] It has been reported that IL-12 and IL-23 are up-regulated in psoriatic skin.[33] Human studies with anti-IL-12p40 have shown that not only this treatment ameliorates psoriasis, but also down-regulates type 1 cytokines and IL-12/IL-23 in lesional skin.[34] Besides sharing the subunit p40 and signaling through similar receptors, IL-23 and IL-12 are responsible for driving different T-cell subsets.

IL-23 could also mediate and sustain late-stage chronic inflammation by the production of IL-17 by Th17.[35] The IL-23/Th17/IL-17 immune axis was initially elucidated when IL-17 gene expression was induced by *B. burgdorferi* independent of IL-12.[36] The IL-17–producing CD4+ T-cells distinct from those producing either IL-4 or IFN-γ were called Th17.[37]

A redundant cytokine model has emerged as the evolving explanation for psoriasis pathogenesis. It is based on the IL-12/Th1/IFN-γ-TNF-α and the IL-23/Th17/IL-17 immune pathways. The effectiveness of the anti-TNF treatment of psoriasis validated the first axis. The efficacy of anti-p40 (anti-IL12/23) treatment confirms the other.[38]

## Selective IL-23/Th17/IL-17 Immune Axis Inhibition

Studies have demonstrated that anti-p40 (anti-IL-12/23) treatment is highly efficacious for psoriasis. Remarkably, in a phase II multicenter, randomized, double-blind, placebo-controlled trial with the human monoclonal antibody anti-IL-12/23 briakinumab, 90-93% of subjects in four dosing groups were able to achieve a PASI 75. This finding along confirms the centrality of this pathway as these levels of efficacy have not been previously seen in studies with other agents.[39] Large registration studies for ustekinumab, also a p40 antibody have also shown substantial and impressive results that place them high in the efficacy hierarchy.[40] Safety data for both agents is limited, but to date has been favorable.

One issue with anti-p40 therapy is that it inhibits both the classical IL-12/Th1/IFN-γ and IL-23/Th17/IL-17 immune pathways. IL-12 and IL-23 are related cytokines with differences in their biological activities. After binding to their receptors, different intracellular transcription complexes are activated.[41] IL-12 predominantly acts on naïve T-cells and initiates the TH1 response. IL-23 primarily affects memory T-cells and expands the initiated Th1 inflammatory response by Th17 activity and maintains an adequate memory pool by compromising memory T-cells.[2,41,42] Experimental studies suggest that IL-23/Th17/IL-17 immune axis blocking is sufficient to treat autoimmune inflammation.[32]

Another way to block both pathways is the immunoregulatory role of IFN-γ. It is well-known that the administration of anti-IFN-γ induces exacerbation of experimental autoimmune encephalomyelitis (EAE).[43] One possible explanation is that the inhibition of the IL-12/Th1/IFN-γ axis may destroy the regulatory role of IFN-γ during chronic inflammation. TNF-α, like INF-γ, has a regulatory role in the immune system.[44] This might explain the observation that anti-TNF therapies induce psoriasis and other autoimmune diseases in some patients.[45]

The increase in efficacy and reduction of adverse events are the main drivers for new therapies. Infections, one type of adverse event, usually increase in patients receiving anti-cytokine therapy.[46] Studies with anti-IL-23 therapy will require surveillance for the development of opportunistic infection. Reports from patients with IL-12 and/or IL-23 cytokine deficiency syndromes alert to these potential infections in individuals under anti-IL-23 therapy. Invasive salmonellosis and mycobacterial diseases were present more often in patients with IL-12/IL-23 deficiency indicating that immunity against these microorganisms appears to be dependent of IL-12 and/or IL-23.[47] However, antibodies against IL-12 and IL-23 may not cause a complete inactivity of these cytokines in a clinical scenario. For example, an experimental study showed that IL-23 plays a role in host defense against *P. carinii*, but it is not an essential one.[48] Clinical studies with anti-IL-12/23 treatment thus far have not increased the risk of non-opportunistic or opportunistic infections.[49] A recent study showed that blocking IL-23 with monoclonal antibodies during BCG infection does not appear to affect the bacterial burden in immunocompetent mice. In contrast, blocking TNF-α or both IL-23 and IL-12 with anti-p40 dramatically enhances micobacterial growth. From this study, antibody blockade of IL-23 alone rather than IL-12 might be preferable in patients who have been or may be exposed to mycobacterial infection.[50]

## Conclusion

A gold standard is the intervention believed to be the best available option. Given the proven role of IL-23 in several models of autoimmune inflammation and psoriasis, substantial interest exists in targeting this cytokine with neutralization immunotherapy. If the IL-23/Th17/IL-17 immune pathway operates in humans as in mice, then specific blockade of the IL-23 immune pathway may be an effective and safer therapy for immune-mediated inflammatory diseases and placing this drug as standard setting paradigm for therapy for psoriasis. However, large studies are needed to provide information on the effects, adverse events of anti-IL-23 therapy and its place in the treatment of psoriasis and other skin diseases.

## References

[1] Feldmann M, Brennan FM, Maini R (1998). Cytokines in autoimmune disorders. Int Rev Immunol.

[2] Oppmann B, Lesley R, Blom B, Timans JC, Xu Y, Hunte B (2000). Novel p19 protein engages IL-12p40 to form a cytokine, IL-23, with biological activities similar as well as distinct from IL-12. Immunity.

[3] Squire B (1873). The etiology of psoriasis. Br Med J.

[4] Heaney JH (1927). The etiology and treatment of psoriasis. Br Med J.

[5] Ingram JT (1953). The approach to psoriasis. Br Med J.

[6] Harber LC, March C, Ovary Z (1962). Lack of passive cutaneous anaphylaxis in psoriasis. Arch Dermatol.

[7] Aswaq M, Farber EM, Moreci AP, Raffel S (1960). Immunologic reactions in psoriasis. Arch Dermatol.

[8] Landau J, Gross BG, Newcomer VD, Wright ET (1965). Immunologic response of patients with psoriasis. Arch Dermatol.

[9] Harris CC (1971). Malignancy during methotrexate and steroid therapy for psoriasis. Arch Dermatol.

[10] Shuster S (1971). Research into psoriasis–the last decade. Br Med J.

[11] Hunter JA, Ryan TJ, Savin JA (1974). Diseases of the skin. Present and future trends in approaches to skin disease. Br Med J.

[12] Braun-Falco O, Christophers E (1974). Structural aspects of initial psoriatic lesions. Arch Dermatol Forsch.

[13] Krueger GG, Jederberg WW, Ogden BE, Reese DL (1978). Inflammatory and immune cell function in psoriasis: II. Monocyte function, lymphokine production. J Invest Dermatol.

[14] Krueger GG, Jederberg WW (1980). Alteration of HeLa cell growth equilibrium by supernatants of peripheral blood mononuclear cells from normal and psoriatic subjects. J Invest Dermatol.

[15] Champion RH (1981). Psoriasis and its treatment. Br Med J (Clin Res Ed).

[16] Bos JD, Hulsebosch HJ, Krieg SR, Bakker PM, Cormane RH (1983). Immunocompetent cells in psoriasis. In situ immunophenotyping by monoclonal antibodies. Arch Dermatol Res.

[17] Baker BS, Swain AF, Griffiths CE, Leonard JN, Fry L, Valdimarsson H (1985). The effects of topical treatment with steroids or dithranol on epidermal T lymphocytes and dendritic cells in psoriasis. Scand J Immunol.

[18] Bos JD, Krieg SR (1985). Psoriasis infiltrating cell immunophenotype: Changes induced by PUVA or corticosteroid treatment in T-cell subsets, Langerhans' cells and interdigitating cells. Acta Derm Venereol.

[19] Lieden G, Skogh M (1986). Plasma exchange and leukapheresis in psoriasis–no effect?. Arch Dermatol Res.

[20] Valdimarsson H, Bake BS, Jónsdótdr I, Fry L (1986). Psoriasis: A disease of abnormal keratinocyte proliferation induced by T lymphocytes. Immunol Today.

[21] Schlaak JF, Buslau M, Jochum W, Hermann E, Girndt M, Gallati H (1994). T-cells involved in psoriasis vulgaris belong to the Th1 subset. J Invest Dermatol.

[22] Mosmann TR, Cherwinski H, Bond MW, Giedlin MA, Coffman RL (1986). Two types of murine helper T-cell clone. I. Definition according to profiles of lymphokine activities and secreted proteins. J Immunol.

[23] Austin LM, Ozawa M, Kikuchi T, Walters IB, Krueger JG (1999). The majority of epidermal T-cells in Psoriasis vulgaris lesions can produce type 1 cytokines, interferon-gamma, interleukin-2, and tumor necrosis factor-alpha, defining TC1 (cytotoxic T lymphocyte) and TH1 effector populations: A type 1 differentiation bias is also measured in circulating blood T-cells in psoriatic patients. J Invest Dermatol.

[24] Schon MP, Boehncke WH (2005). Psoriasis. N Engl J Med.

[25] Nickoloff BJ, Schroder JM, von den Driesch P, Raychaudhuri SP, Farber EM, Boehncke WH (2000). Is psoriasis a T-cell disease?. Exp Dermatol.

[26] Okamura H, Tsutsi H, Komatsu T, Yutsudo M, Hakura A, Tanimoto T (1995). Cloning of a new cytokine that induces IFN-gamma production by T-cells. Nature.

[27] Biedermann T, Rocken M, Carballido JM (2004). TH1 and TH2 lymphocyte development and regulation of TH cell-mediated immune responses of the skin. J Investig Dermatol Symp Proc.

[28] Macatonia SE, Hosken NA, Litton M, Vieira P, Hsieh CS, Culpepper JA (1995). Dendritic cells produce IL-12 and direct the development of Th1 cells from naive CD4+T-cells. J Immunol.

[29] Trinchieri G (1998). Interleukin-12: A cytokine at the interface of inflammation and immunity. Adv Immunol.

[30] Hong K, Berg EL, Ehrhardt RO (2001). Persistence of pathogenic CD4+ Th1-like cells in vivo in the absence of IL-12 but in the presence of autoantigen. J Immunol.

[31] Hong K, Chu A, Ludviksson BR, Berg EL, Ehrhardt RO (1999). IL-12, independently of IFN-gamma, plays a crucial role in the pathogenesis of a murine psoriasis-like skin disorder. J Immunol.

[32] Monteleone I, Pallone F, Monteleone G (2009). Interleukin-23 and Th17 cells in the control of gut inflammation. Mediators Inflamm.

[33] Lee E, Trepicchio WL, Oestreicher JL, Pittman D, Wang F, Chamian F (2004). Increased expression of interleukin 23 p19 and p40 in lesional skin of patients with psoriasis vulgaris. J Exp Med.

[34] Toichi E, Torres G, McCormick TS, Chang T, Mascelli MA, Kauffman CL (2006). An anti-IL-12p40 antibody down-regulates type 1 cytokines, chemokines, and IL-12/IL-23 in psoriasis. J Immunol.

[35] Aggarwal S, Ghilardi N, Xie MH, de Sauvage FJ, Gurney AL (2003). Interleukin-23 promotes a distinct CD4 T-cell activation state characterized by the production of interleukin-17. J Biol Chem.

[36] Infante-Duarte C, Horton HF, Byrne MC, Kamradt T (2000). Microbial lipopeptides induce the production of IL-17 in Th cells. J Immunol.

[37] Harrington LE, Hatton RD, Mangan PR, Turner H, Murphy TL, Murphy KM (2005). Interleukin 17-producing CD4+ effector T-cells develop via a lineage distinct from the T helper type 1 and 2 lineages. Nat Immunol.

[38] Nestle FO, Kaplan DH, Barker J (2009). Psoriasis. N Engl J Med.

[39] Lima XT, Abuabara K, Kimball AB, Lima HC (2009). Briakinumab. Expert Opin Biol Ther.

[40] Leonardi CL, Kimball AB, Papp KA, Yeilding N, Guzzo C, Wang Y (2008). Efficacy and safety of ustekinumab, a human interleukin-12/23 monoclonal antibody, in patients with psoriasis: 76-week results from a randomised, double-blind, placebo-controlled trial (PHOENIX 1). Lancet.

[41] Parham C, Chirica M, Timans J, Vaisberg E, Travis M, Cheung J (2002). A receptor for the heterodimeric cytokine IL-23 is composed of IL-12Rbeta1 and a novel cytokine receptor subunit, IL-23R. J Immunol.

[42] Trinchieri G, Pflanz S, Kastelein RA (2003). The IL-12 family of heterodimeric cytokines: New players in the regulation of T-cell responses. Immunity.

[43] Becher B, Durell BG, Noelle RJ (2002). Experimental autoimmune encephalitis and inflammation in the absence of interleukin-12. J Clin Invest.

[44] Liu J, Marino MW, Wong G, Grail D, Dunn A, Bettadapura J (1998). TNF is a potent anti-inflammatory cytokine in autoimmune-mediated demyelination. Nat Med.

[45] Ramos-Casals M, Brito-Zeron P, Soto MJ, Cuadrado MJ, Khamashta MA (2008). Autoimmune diseases induced by TNF-targeted therapies. Best Pract Res Clin Rheumatol.

[46] Dinarello CA (2003). Anti-cytokine therapeutics and infections. Vaccine.

[47] MacLennan C, Fieschi C, Lammas DA, Picard C, Dorman SE, Sanal O (2004). Interleukin (IL)-12 and IL-23 are key cytokines for immunity against Salmonella in humans. J Infect Dis.

[48] Rudner XL, Happel KI, Young EA, Shellito JE (2007). Interleukin-23 (IL-23)-IL-17 cytokine axis in murine Pneumocystis carinii infection. Infect Immun.

[49] Shear NH, Prinz J, Papp K, Langley RG, Gulliver WP (2008). Targeting the interleukin-12/23 cytokine family in the treatment of psoriatic disease. J Cutan Med Surg.

[50] Chackerian AA, Chen SJ, Brodie SJ, Mattson JD, McClanahan TK, Kastelein RA (2006). Neutralization or absence of the interleukin-23 pathway does not compromise immunity to mycobacterial infection. Infect Immun.

